# Images of colonic real-time tissue sonoelastography correlate with those of colonoscopy and may predict response to therapy in patients with ulcerative colitis

**DOI:** 10.1186/1471-230X-11-29

**Published:** 2011-03-31

**Authors:** Daisuke Ishikawa, Takafumi Ando, Osamu Watanabe, Kazuhiro Ishiguro, Osamu Maeda, Nobuyuki Miyake, Masanao Nakamura, Ryoji Miyahara, Naoki Ohmiya, Yoshiki Hirooka, Emad M El-Omar, Hidemi Goto

**Affiliations:** 1 Department of Gastroenterology, Nagoya University Graduate School of Medicine, Nagoya, Japan; 2 Department of Endoscopy, Nagoya University Hospital, Nagoya, Japan; 3 Department of Medicine and Therapeutics, University of Aberdeen, Aberdeen, UK

## Abstract

**Background:**

Real-time tissue sonoelastography (EG) is a new non-invasive technique that visualizes differences in tissue strain. We evaluated the usefulness of EG in patients with ulcerative colitis (UC) by investigating the association between EG and colonoscopic findings and disease activity.

**Methods:**

Thirty-seven UC patients undergoing EG and colonoscopy were invited to enroll. EG findings were classified as normal, homogeneous, random, or hard, and colonoscopic findings as normal, mucosal edema and erosion, punched-out ulcer, and extensive mucosal abrasion. Clinical findings were evaluated using clinical activity index (CAI) scores for each patient at colonoscopy.

**Results:**

On EG, 10 cases were classified as normal, 11 as homogeneous, 6 as random, and 10 as hard. EG findings showed a significant correlation those of colonoscopy (*p *< 0.001). Seven of 10 (70%) normal-type patients were in the remission phase, while all 6 random-type patients were in the active phase. Among active-phase patients, 4 of 7 (57%) homogeneous-type patients responded to steroid or leukocytapheresis therapy, while 3 of 6 (50%) random-type patients required treatment with cyclosporine. Three of 10 (30%) hard-type patients required colectomy.

**Conclusions:**

In this small series, EG findings reflected colonoscopic findings and correlated with disease activity among patients with UC.

## Background

Although colonoscopic examination is important in determining the treatment of UC, it is an invasive procedure which can lead to complications. Non-invasive methods of evaluating the large bowel in patients with UC have therefore been sought. One candidate is transabdominal ultrasonography (US), a useful and non-invasive diagnostic procedure which is chiefly used in the examination of hepatobiliary and pancreatic diseases. The recent marked improvement in ultrasound devices now allows the use of US in the diagnosis of gastrointestinal disorders [1,2], including inflammatory bowel disease (IBD) [3-6]. However, the role of US in the diagnosis of disease activity in these conditions is unknown, leaving colonoscopy as the standard procedure for this purpose, its invasiveness notwithstanding.

Real-time tissue sonoelastography (EG) is a new technique that visualizes the differences in tissue strain produced by freehand compression during routine US. In EG, pressure is applied to the tissue, and the resulting difference in distortion between hard and soft tissues is used to visualize the hardness of various tissues in real-time. Images obtained by EG thus provide information on tissue elasticity, and in turn reflect histopathologic differences. EG has been used for the diagnosis of focal lesions in superficial organ lesions, such as in the breast and thyroid gland [7-9]. EG has also been reported to be useful in the evaluation of lymph nodes and the diagnosis of pancreatic and liver diseases [10,11]. Recently, the application of EG to lesions of the gastrointestinal tract has been also attempted. To demonstrate its feasibility in intestinal fibrosis, for example, Kim et al. used EG in an animal model of IBD, and suggested the feasibility of translating this imaging technique directly to human subjects for both diagnosis and monitoring [12].

Here, we conducted a single-center, retrospective and prospective study to investigate whether differences in tissue strain of the mucosal layer obtained by EG appropriately reflect colonoscopic findings and correlate with disease activity among patients with UC.

## Methods

### Subjects

From March 2006 to November 2007, 37 consecutive patients with UC (16 males and 21 females, age 44.3 ± 16.2y) undergoing colonoscopy at our institution were invited to enroll (Table 1). Patients with proctitis only were excluded. Twenty-eight patients had relapsing-remitting disease and nine had chronic continuous disease. With regard to disease extension, 17 cases were left-sided colitis and 20 were the pan-colitis type. All subjects gave written informed consent, and the study was approved by the Ethics Committee of Nagoya University Graduate School of Medicine.

**Table 1:** Patient characteristics

Characteristic	
Age in years (range)	44.3 ± 16.2 (24-79)
Sex	
male, (%)	16 (43.2)
female, (%)	21 (56.8)
Duration of disease in months (range)	63.2 ± 33.2
Type of disease	
relapsing-remitting, (%)	28 (75.7)
chronic continuous, (%)	9 (24.3)
Location of colitis	
pancolitis, (%)	20 (54.1)
left-sided colitis, (%)	17 (45.9)
Smoking habit, (%)	8 (21.6)

### Real-time Tissue Elastography (EG)

EG was conducted using a Hitachi EUB-8500 US system (Hitachi Medical, Tokyo, Japan). In this system, the object is observed using a linear probe (EUP-L52, 3.5Y7.5 MHz), with B- and EG-mode images simultaneously visualized on a dual screen. For EG, B-mode and EG-mode images were simultaneously visualized on a dual screen. The region of interest (ROI) was arbitrarily configured in the EG-mode image, and elasticity distribution within it was represented in different colors (soft, red; hard blue) by freehand compression with the ultrasound probe. A phantom model consisted of two types of gel, in which a harder gel-shaped column was inserted into a soft gel. The columnar gel was not visible in the B-mode image, but was visible in the EG-mode image owing to the difference in elasticity between them. The principal of EG can be explained in terms of a one-dimensional spring model [13]. This model is composed of hard and soft springs that couple together one-dimensionally: when compressed, the soft spring is highly deformed, whereas the hard spring is hardly deformed. The amount of distortion, calculated by differentiating the displacement variation of the spring, provides information on elasticity.

### Clinical procedures

EG was performed with the EUB-8500 US just before colonoscopy, after ingestion of an oral electrolyte lavage solution. Because EG requires compression of the target organ, we considered that the descending colon was suitable for scanning because it is fixed to the retroperitoneum. Compressing the abdominal wall with the ultrasound probe also compressed the descending colon, and EG-mode images of the descending colon were clearly obtained. Total colonoscopy was performed after the EG examination was finished, with careful observation of the mucosa of the descending colon. EG was performed by a single physician who specializes in the EG procedure, while colonoscopy was performed by two endoscopists.

### Classification of EG findings

**Figure 1:**
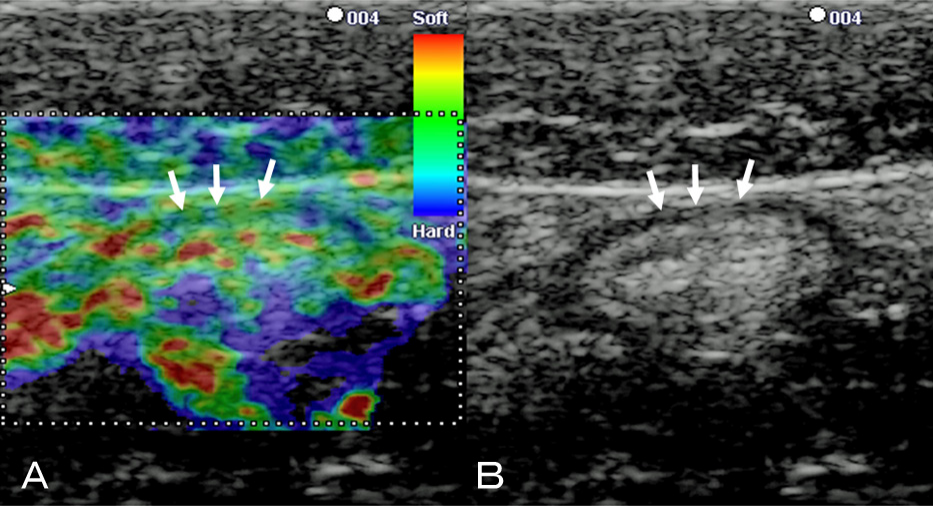
**B-mode ultrasound and corresponding EG images of the normal colon wall**. A: Corresponding EG of the normal wall shows almost all parts of the wall appear green (arrows)..B: B-mode ultrasound image of the normal colon wall shows 5 layers (arrows).

**Figure 2:**
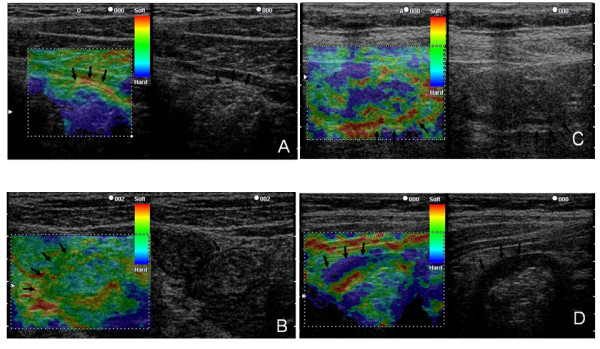
**Classification of elastography findings**. A: Normal type: the wall of the colon was not thickened (less than 4 mm), and the layer structure was observed in B-mode. Almost all parts of the wall appeared green in EG-mode, but the second layer was sometimes red. B: Homogeneous type: the wall was thick in B-mode but the layer structure was unclear. The thick wall was almost completely green in EG-mode. C: Random type: the wall was thick in B-mode and the layer structure was unclear. Various colors (red, green, blue) were mixed throughout the thick wall in EG-mode. D: Hard type: the wall was thick in B-mode and the layer structure was unclear. The thick wall was almost completely blue.

Images of the descending colon obtained in EG-mode were classified into four types (normal, homogeneous, random, hard) on the basis of color arrangement. For the normal type, the wall of the colon was not thick (less than 4 mm) and a five-layer structure was present in B-mode (Figure 1B). Almost all parts of the wall of this type were green (Figure 1A). For the other types (homogeneous, random, and hard), the wall of the colon was thickened and the layer structure was unclear in B-mode because of inflammation [14]. We recognized three types of color arrangement in the thick wall in EG-mode: a homogeneous type, in which the thick wall was nearly completely green; a random type, in which the thick wall was imaged in various colors (red, green, blue); and a hard type, in which the thick wall was nearly completely blue (Figure 2).

### Classification of colonoscopic findings

**Figure 3:**
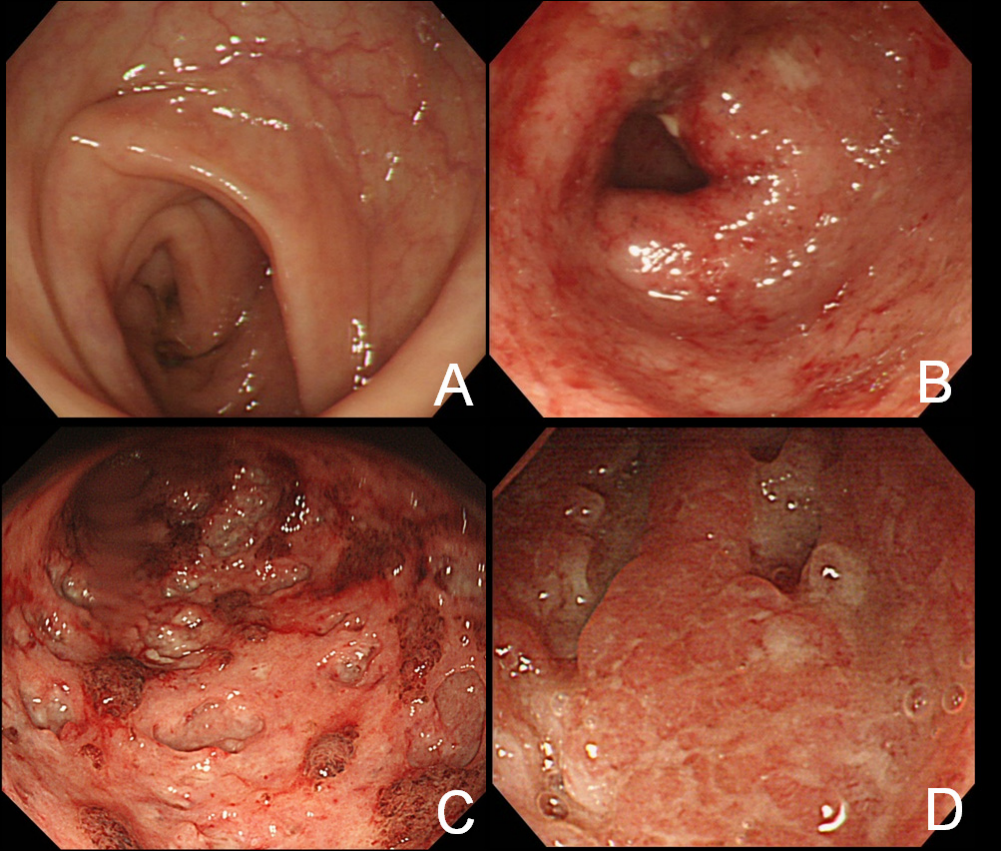
**Classification of colonoscopy findings**. A: Normal mucosa without erosion or ulcer. B: Mucosal edema and erosion without ulcer. C: Punched-out ulcer. D: Extensive ulcer.

Endoscopy findings were classified into four types by the presence and extent of ulcers, as follows: Type A, normal mucosa without erosion or ulcer; B, mucosal edema and erosion without ulcer; C, punched-out ulcer; and D, extensive ulcer (Figure 3).

### Clinical activity index

Clinical findings were evaluated using clinical activity index (CAI) scores for each patient at colonoscopy [15], which were based on eight clinical parameters: diarrhea (0-4), nocturnal diarrhea (0-1), visible blood in stool (0-3), fecal incontinence (0-1), abdominal pain or cramping (0-3), general well-being (0-5), abdominal tenderness (0-3), and need for antidiarrheal drugs (0-1), with a maximum score of 21.

### Statistical analysis

All data values are shown as means ± S.E. Statistical analysis was performed using the chi-square test for independence. Differences with P values less than .05 were considered significant.

## Results

### EG and colonoscopic findings

**Figure 4:**
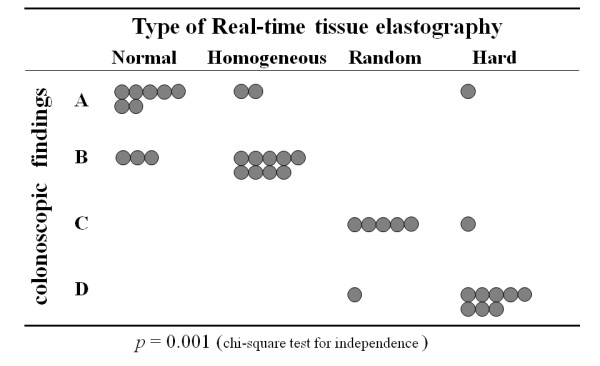
**Relationship between the findings of real-time tissue elastography and colonoscopy**. There are significant associations between EG and colonoscopic findings (*p *< 0.001).

Application of the US ventrally to image the ventral part of the colon obviated the air barrier to US transmission, and good EG- and B-mode images were obtained in all patients. On EG, 10 cases were classified as normal, 11 as homogeneous, 6 as random, and 10 as hard; while on endoscopy, 10 were classified as type A, 12 as type B, 6 as type C, and 9 as type D. We found significant associations between EG and colonoscopic findings (*p *< 0.001), namely between the normal type and type A, homogeneous type and type B, random type and type C, and hard type and type D (Figure 4).

### EG findings and CAI assessment

We then compared the relationship between EG finding and clinical activity. Of 37 UC patients, 16 were in the remission phase and 21 were in the active phase. Seven of 10 patients (70%) with the normal type were in remission, while 7 of 11 (64%) with the homogeneous type, 6 of 6 (100%) with the random type, and 5 of 10 (50%) with the hard type were in the active phase (Table 2). Individuals in the active phase were more likely to show elastographic findings other than "normal" (*p *< 0.046).

**Table 2:** Elastographic findings and clinical classification

	Normal	Homogeneous	Random	Hard	total
Remission phase	7 (44%)	4 (25%)	0 (0)	5 (31%)	16
Active phase	3 (14%)	7 (33%)	6 (29%)	5 (24%)	21

*p *= 0.046 (Fisher's exact test for homogeneity)

## Discussion

In this study, we found that EG findings in patients with UC reflected colonoscopic findings and correlated with patient outcome. These findings suggest that EG may represent a useful approach in determining the optimal treatment for UC.

Technologic advances in US and accumulated experience in the interpretation of images mean that US can now yield substantial information about gastrointestinal disorders [1]. The diagnosis of lower alimentary tract conditions by transabdominal US has been widely investigated. Among several studies of inflammatory bowel disease, Maconi et al. reported that US-determined bowel wall thickness correlated with the clinical and endoscopic activity of UC [5]; while Hata et al. classified US images into four types by bowel wall thickness and stratification, and concluded that the association of these types with both disease activity and endoscopic findings made transabdominal US useful in the evaluation of inflammatory changes in the bowel [4].

We reasoned that EG-mode could provide new information on the elasticity of the inflamed colon, in addition to that on wall thickness and stratification observed as in B-mode, and that the diagnostic value of evaluating inflammation of the colon with EG-mode would compare favorably with that by colonoscopy. Here, we set the ROI to include the area that surrounded the bowel, which appeared red in EG-mode. We considered this tissue to be circumintestinal adipose tissue, and used it as a benchmark of elasticity of the bowel. Bowel wall which was of the normal type in EG-mode was not thickened in B-mode, and the layer structure was clearly observed. In contrast, the bowel wall of the other EG-mode types (homogeneous, random, and hard types) was thickened and lacked stratification. The wall of the EG-mode random type was variously colored red, green and blue, suggesting that this type of wall had highly variable elasticity. Endoscopic findings with the EG-mode random type were a punched-out ulcer or extensive ulcer. On the basis of biopsy findings, we speculated that the base of these ulcers may be hard and the mucosa around them may be soft. The wall of the EG-mode homogeneous type was nearly completely green. Endoscopic findings with this type included edema and erosion, but not ulcer. We speculated that the edematous bowel wall or erosions occurred as a soft image in EG-mode. The thickened wall of the EG-mode hard type was blue. Endoscopic findings for this type differed depending on clinical phase: active-phase patients showed extensive ulceration and wide mucosal defects, whereas some patients in remission showed atrophic mucosa without ulceration. We considered that strong and persistent inflammation of the mucosa might have resulted in an atrophic appearance and caused fibrosis in the bowel wall, and speculated that the fibrotic bowel wall occurred as a hard image in EG-mode. Rates of remission in EG-mode normal, homogeneous, random, and hard type cases were 70%, 36%, 0%, and 50%, respectively. Allowing for the fact that this is an early finding in a small series, individuals in the active phase appeared more likely to show elastographic findings other than "normal" (*p *< 0.046). The drawing of any conclusive association between EG findings and CAI assessment awaits further study.

Several limitations of the present study warrant mention. First, the patient population was small (n = 37), and confirmation in a larger number of patients is required. Second, all patients enrolled were from our institution, indicating the possibility of participation bias. Future studies should be conducted in a larger number of patients from various institutions. Third, EG was performed by one physician who specialized in the EG procedure. Generalization of EG assessment requires the analysis of interobserver variability among physicians.

## Conclusions

EG appropriately reflects colonoscopic findings and may be useful in assessing disease activity. Effective clinical use of EG requires additional study.

## Abbreviations

EG: elastography; UC: ulcerative colitis; US: ultrasonography; IBD: inflammatory bowel disease; CAI: clinical activity index; G-CAP: granulocytapheresis; L-CAP: leukocytapheresis; CyA: cyclosporine, ROI: region of interest

## Competing interests

The authors declare that they have no competing interests.

## Authors' contributions

DI, TA, OW, and HG were responsible for the study design and co-ordination. Samples were collected and prepared by DI, TA, and OW. Elastography was performed by DI, TA, and OW. DI analyzed the data. TA drafted the report. All authors participated in critical version of the report. All authors read and approved the final version of the manuscript.

## Pre-publication history

The pre-publication history for this paper can be accessed here:

http://www.biomedcentral.com/1471-230X/11/29/prepub
